# Strain patterns in normal anterior talofibular and calcaneofibular ligaments and after anatomical reconstruction using gracilis tendon grafts: A cadaver study

**DOI:** 10.1186/s12891-021-04444-w

**Published:** 2021-06-18

**Authors:** Masato Takao, Danielle Lowe, Satoru Ozeki, Xavier M. Oliva, Ryota Inokuchi, Takayuki Yamazaki, Yoshitaka Takeuchi, Maya Kubo, Kentaro Matsui, Mai Katakura, Mark Glazebrook

**Affiliations:** 1 Clinical and Research Institute for Foot and Ankle Surgery, 341-1, Mangoku, Kisarazu, Chiba, 292-0003 Japan; 2 Department of Orthopaedic Surgery, Lions Gate Hospital, North Vancouver, BC Canada; 3 Department of Orthopaedic Surgery, Dokkyo Medical University Saitama Medical Center, 2-1-50, Minamikoshigaya, Koshigaya, Saitama, Japan; 4 Department of Human Anatomy, University of Barcelona, Calle Casanova, 143, 08038 Barcelona, Spain; 5 Department of Health Services Research, Faculty of Medicine, University of Tsukuba, 1-1-1 Tenno-dai, Tsukuba, Ibaraki, Japan; 6 Tokyo National College of Technology, 1220-2, Kunugida-machi, Hachioji, Tokyo, Japan; 7 Department of Orthopaedic Surgery, Teikyo University, 2-11-1 Kaga, Itabashi, Tokyo, Japan; 8 Department of Joint Surgery and Sports Medicine, Tokyo Medical and Dental University, 1-5-45 Yushima, Bunkyo, Tokyo, Japan; 9 Division of Orthopaedic Surgery, Dalhousie University, Queen Elizabeth II Health Sciences Center Halifax Infirmary (Suite 4867), 1796 Summer Street, Halifax, NS B3H3A7 Canada

**Keywords:** Anterior talofibular ligament, Calcaneofibular ligament, Miniaturization ligament performance probe, Tension

## Abstract

**Background:**

Inversion ankle sprains, or lateral ankle sprains, often result in symptomatic lateral ankle instability, and some patients need lateral ankle ligament reconstruction to reduce pain, improve function, and prevent subsequent injuries. Although anatomically reconstructed ligaments should behave in a biomechanically normal manner, previous studies have not measured the strain patterns of the anterior talofibular ligament (ATFL) and calcaneofibular ligament (CFL) after anatomical reconstruction. This study aimed to measure the strain patterns of normal and reconstructed ATFL and CFLs using the miniaturization ligament performance probe (MLPP) system.

**Methods:**

The MLPP was sutured into the ligamentous bands of the ATFLs and CTLs of three freshly frozen cadaveric lower-extremity specimens. Each ankle was manually moved from 15° dorsiflexion to 30° plantar flexion, and a 1.2-N m force was applied to the ankle and subtalar joint complex.

**Results:**

The normal and reconstructed ATFLs exhibited maximal strain (100) during supination in three-dimensional motion. Although the normal ATFLs were not strained during pronation, the reconstructed ATFLs demonstrated relative strain values of 16–36. During the axial motion, the normal ATFLs started to gradually tense at 0° plantar flexion, with the strain increasing as the plantar flexion angle increased, to a maximal value (100) at 30° plantar flexion; the reconstructed ATFLs showed similar strain patterns. Further, the normal CFLs exhibited maximal strain (100) during plantar flexion-abduction and relative strain values of 30–52 during dorsiflexion in three-dimensional motion. The reconstructed CFLs exhibited the most strain during dorsiflexion-adduction and demonstrated relative strain values of 29–62 during plantar flexion-abduction. During the axial motion, the normal CFLs started to gradually tense at 20° plantar flexion and 5° dorsiflexion.

**Conclusion:**

Our results showed that the strain patterns of reconstructed ATFLs and CFLs are not similar to those of normal ATFLs and CFLs.

## Background

Inversion sprains of the lateral ankle ligaments may result in symptomatic lateral ankle instability that is resistant to treatment in approximately 20% of patients [1–5]. Further, persistent lateral ankle instability may lead to osteochondral lesions of the talar dome [6, 7] and eventually result in osteoarthritis of the ankle [8]. Moreover, the pain and instability associated with compromised ankle biomechanics may decrease the patient’s participation in common activities of daily living and sports performance [9]. Therefore, lateral ankle ligament repair or reconstruction may be indicated to reduce pain, improve function, and prevent subsequent injuries should more conservative treatment measures fail.

When performing corrective surgery, a reconstruction technique is indicated if the ligament is absent or insufficient residual ligament is available for reconstruction [10, 11]. Anatomic reconstruction of the lateral ankle ligaments using tendon grafts is the most commonly used surgical technique [12, 13], and it has evolved to become a minimally invasive arthroscopic procedure [14–18]. In this technique, bone tunnels are made at the anatomic origin and insertion points of the ligament, and tendon grafts are then introduced and fixed. Although the anatomically reconstructed ligament is assumed to behave in a biomechanically normal manner, there have not been any publications documenting this assumption.

In this study, the miniaturization ligament performance probe (MLPP) system [19] was used to determine the strain patterns in anatomically reconstructed anterior talofibular ligaments (ATFLs) and calcaneofibular ligaments (CFLs), and the strain patterns were compared with those of normal ATFL and CFL.

## Methods

### Cadaver description

Three freshly frozen cadaveric through-the-knee lower-extremity specimens (two right and one left) were used for this study. The median age of the cadavers at the time of death was 63 years (range, 52–70 years); one specimen was from a male and the others from females. The specimens were free of ankle or hind foot deformities, had not undergone prior surgeries or dissections, and did not have histories of trauma or other pathologies that may have altered their anatomy. The cadaveric studies were performed at the University of Barcelona (Spain); the methods were reviewed and approved by the university’s Institutional Review Board. Informed consent for the storage and use of the bodies for research purposes was given by the donors prior to their deaths or by their next of kin.

### Strain pattern determinations in normal ATFLs and CFLs

**Fig. 1:**
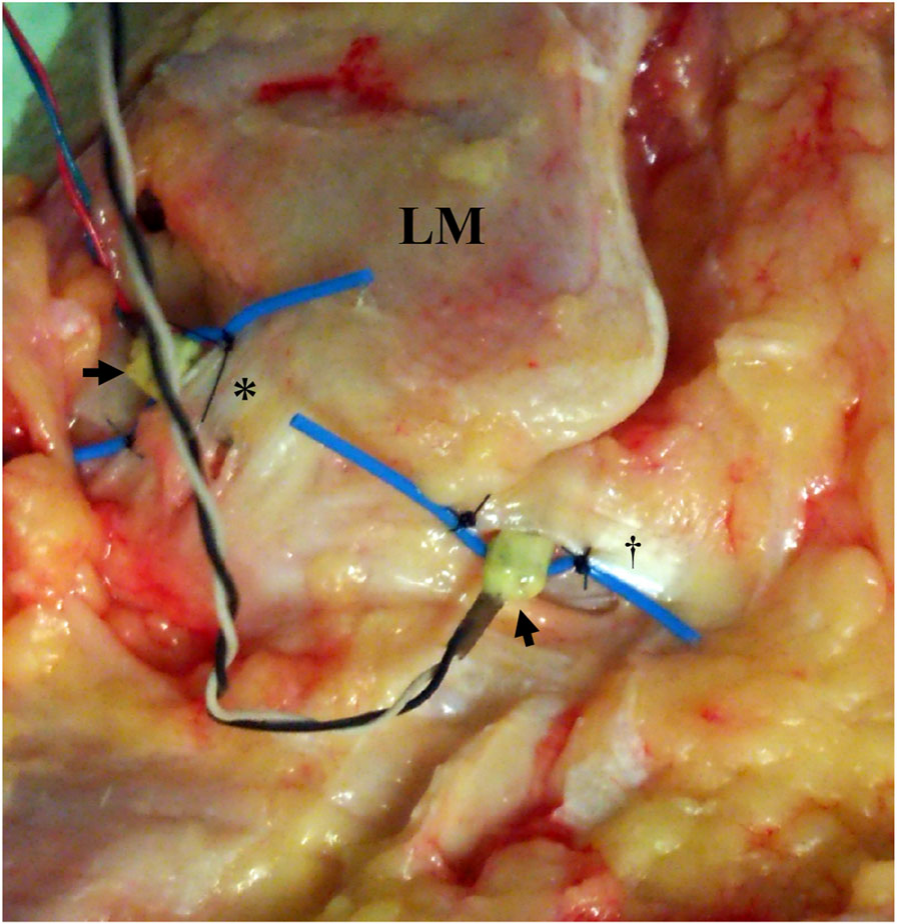
Force probes in normal anterior talofibular ligament (ATFL) and calcaneofibular ligament (CFL). A force probe (arrow) is shown inserted into the mid-substance of each ligamentous band of the ATFL (*) and CFL (†)

In all specimens, dissections and subsequent strain measurements were performed by one experienced foot and ankle surgeon. An incision was made on the lateral ankle and the ATFL and CFL were exposed. This procedure has been described in detail in our previous study [20]. As the ligaments were investigated as a single unit, they were not isolated after exposure. A force probe was placed into the mid-substance of each ligamentous band of the ATFL and CFL, such that the force probe slit was aligned with the long axis of the ligament fibers (Fig. 1). After introducing the force probe into the ligament, the force probe tube was sutured to the ligament fibers with a 3-0 nylon thread to prevent its rotation [19].

**Fig. 2:**
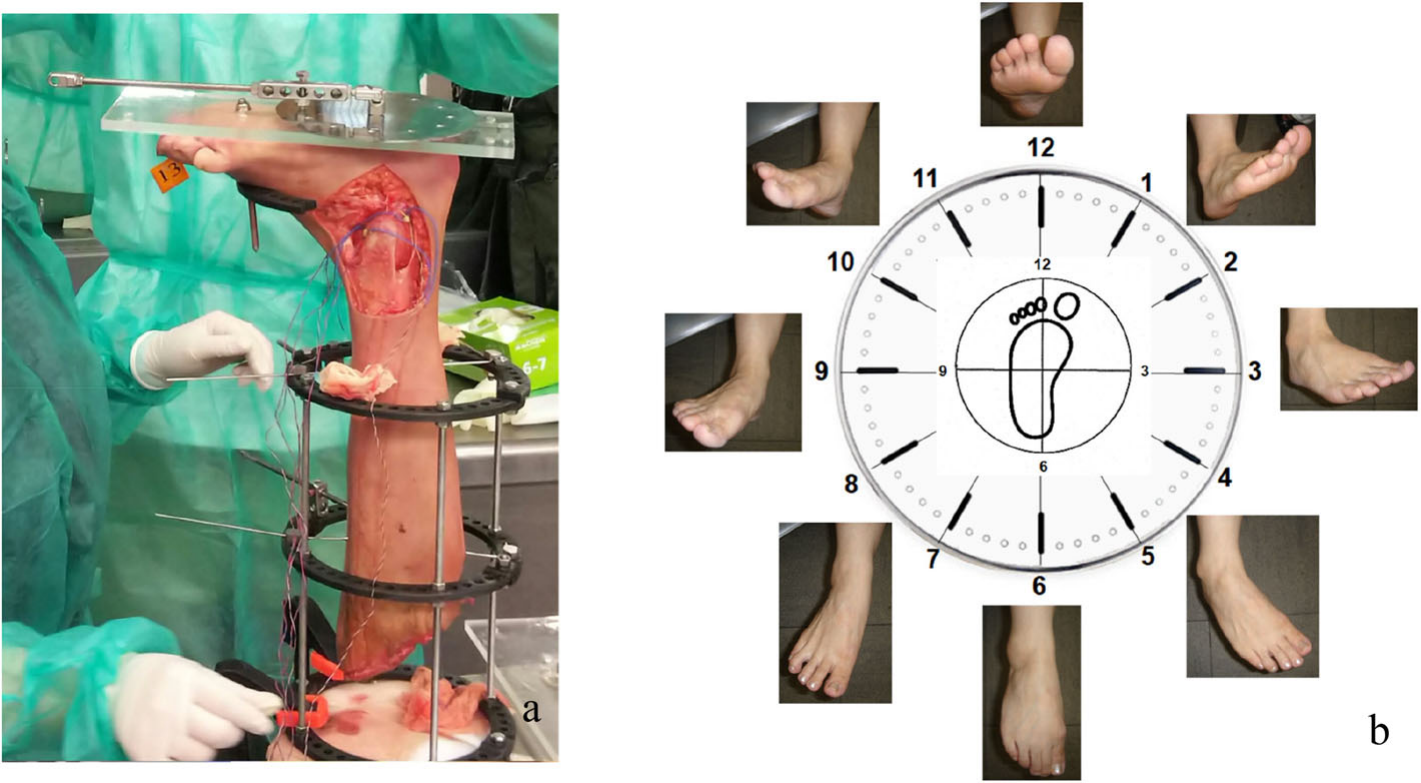
Clock plate set up for measurements. **a** A round metal disk (clock; diameter, 150 mm) with 6-mm-diameter holes placed every 30° around the perimeter is shown affixed to an acrylic plate. The acrylic plate is fixed to the plantar aspect of the foot, using a screw inserted into the calcaneus and a rod inserted between the second and third metatarsals. **b** The ankle positions were defined as dorsiflexion when the arm was in the 12 o’clock position, plantar flexion (6 o’clock position), inversion (3 o’clock position), and eversion (9 o’clock position); in addition, the 1 and 2 o’clock positions were defined as dorsiflexion-adduction, 4 and 5 o’clock positions represented supination (plantar flexion-adduction), 7 and 8 o’clock positions represented plantar flexion-abduction, and the 10 and 11 o’clock positions were defined as pronation (dorsiflexion-abduction)

An Ilizarov ring-shaped external fixator was placed on the lower leg, and the lower limb was vertically fixed to the measurement desk. A round metal disk (called a “clock”; diameter, 150 mm) with 6-mm-diameter holes placed every 30° around the clock perimeter was affixed to an acrylic plate (width, 120 mm; length, 280 mm; thickness, 10 mm). Then, the plate was fixed to the plantar aspect of the foot with a screw (diameter, 6 mm) inserted into the calcaneus and a rod (diameter, 8 mm) inserted between the second and third metatarsals (Fig. 2a). This plate had a 25-cm arm to which a 0.5-kg weight could be added to the free end, applying a 1.2-N m force to the ankle and subtalar joint complex (0.5 kg × 0.25 m × 9.81 = 1.23 N m) [19].

The arm was rotated in 30° increments to allow strain measurements of each ligamentous band at different ankle positions. The ankle positions were defined as dorsiflexion when the arm was in the 12 o’clock position, plantar flexion when in the 6 o’clock position, inversion when in the 3 o’clock position, and eversion when in the 9 o’clock position. In addition, the 1 and 2 o’clock positions were defined as dorsiflexion-adduction, 4 and 5 o’clock positions represented supination (plantar flexion-adduction), 7 and 8 o’clock positions represented plantar flexion-abduction, and 10 and 11 o’clock positions represented pronation (dorsiflexion-abduction) (Fig. 2b), according to the terminology proposed by the Ad Hoc Committee of Terminology of the Japanese Society for Surgery of the Foot [21].

After determining the strain in each designated ankle position, the strain values of each ankle ligament were also measured during axial motion of the ankle from 15° dorsiflexion to 30° plantar flexion. The angles of the axial, sagittal, and horizontal motions were measured using an electronic goniometer (MPU-9250; TDK InvenSense, San Jose, CA, USA) synchronized to the MLPP system [19].

### Strain pattern determinations in the reconstructed ATFLs and CFLs

**Fig. 3:**
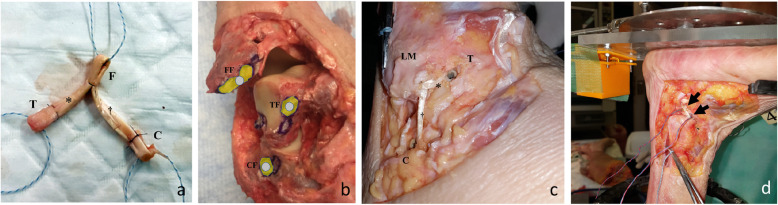
Anterior talofibular ligament (ATFL) and calcaneofibular ligament (CFL) reconstruction. **a** Anatomic Y-configuration tendon graft. The graft is prepared in an anatomic “Y” configuration with graft loops at the three ends (*F* fibular limb, *T* talar limb, *C* calcaneal limb) of the anatomic Y-grafts of the ATFL (*) and CFL (†). **b** Footprints (*FF* fibular footprint, *TF* talar footprint, *CF* calcaneal footprint) and position of each bone tunnel (gray circle). **c** Reconstructed ATFL (*) and CFL (†). **d** Force probes (arrow) inserted into the normal ATFL and CFL. *LM* lateral malleolus, *T* talus, *C* calcaneus

For each cadaveric limb, after determining the strain patterns of normal ATFLs and CFLs, these ligaments were removed at their origin and insertion points to identify their anatomic footprints. The ATFL and CFL reconstructions were performed by a technique similar to that previously used actual patients for patients with chronic lateral instability [18]. An gracilis tendon autograft (approximately 135 mm long) was harvested from the cadaver’s ipsilateral knee. The graft was prepared in an anatomic “Y” configuration with graft loops at the three ends of the anatomic Y-graft to facilitate suture attachment and graft delivery, as previously described [18] (Fig. 3a).

A guide wire was inserted into the centers of the fibular ATFL and CFL footprints on the fibular obscure tubercle and directed toward the proximal and posterior edges of the fibula at an angle of approximately 30° up to the long axis of the fibula, allowing the guide wire to pass through the central portion of the fibula on the coronal axis. Then, the guide wire was passed though the posterior cortex of the fibula and through the skin, posterior to the fibula. Over drilling (diameter, 6 mm) was then performed up to a depth of 20 mm. A talar tunnel was constructed to serve as the docking site for the talar stem of the anatomic Y-graft. Next, a guide wire was inserted to penetrate the talus through the center of the ATFL insertion footprint and directed toward the distal end of the medial malleolus. The guide wire was passed through the medial wall of the talus and then through the skin, just anterior and slightly distal to the tip of the medial malleolus. Over drilling was again performed.

Further, a calcaneal bone tunnel was also constructed to serve as the docking site for the calcaneal stem of the anatomic Y-graft. A guide wire was used to penetrate the calcaneal CFL insertion site footprint and directed toward the central posterior cortex of the calcaneus. The guide wire was then over drilled (diameter, 6 mm) to a depth of 30 mm. The three anatomic Y-graft stems were then inserted into their respective tunnels up to a depth of at least 15 mm and fixed with interference screws. Each bony attachment of the tendon graft was fixed with a 6-mm-diameter interference screw while applying a 30-N tension force. The fibular stem was fixed and then the talar stem was fixed while the ankle was in a 0° flexed neutral position. The calcaneal attachment was fixed in a similar manner (Fig. 3b).

After reconstruction of the ATFLs and CFLs, the strain values of the reconstructed ligaments were measured using the MLPP system during axial and three-dimensional motion, similar to the normal ATFL and CFL investigations, as previously described (Fig. 3c, d) [19].

### Data analysis

The relationships between the foot positions and the tensile forces of each ligamentous band were analyzed. The tensile force data from the force probe were obtained by synchronizing the arm of the clock, which was manually rotated every 30°, with the movement of the ankle from 15° dorsiflexion to 30° plantar flexion; the movements were repeated 10 times (for each limb) and the strain of each ligamentous band as measured during ankle motion. Individual strain data were aligned to reflect the strain relative to the neutral position (0) and to the maximal strain value (100). The average value determined at each position was connected by a line, and the ligament tension patterns were compared between the normal and reconstructed specimens [19].

## Results

### Strain patterns in normal and reconstructed ATFLs

**Fig. 4:**
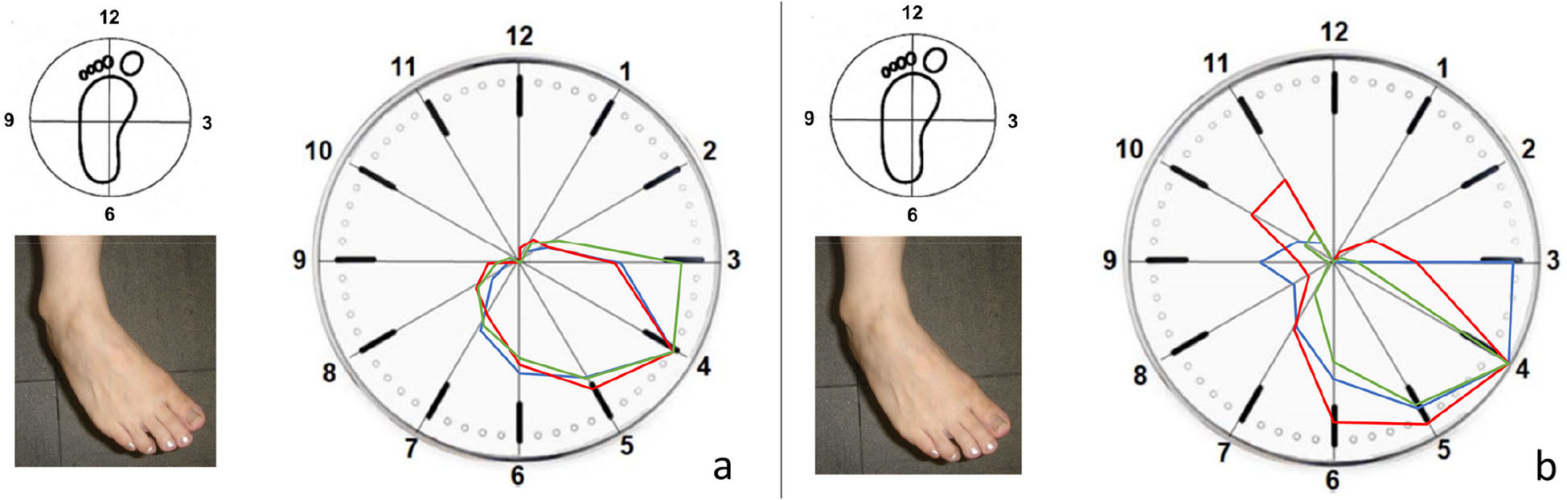
Strain patterns of normal and reconstructed anterior talofibular ligaments (ATFLs) during three-dimensional motion. **a** The normal ATFL exhibited the maximal strain value (strain value, 100) during supination. **b** The reconstructed ATFL also exhibited the maximal strain value (100) during supination. Although the normal ATFL does not exhibit strain during pronation, the reconstructed ATFL shows strain values of 16–36 during pronation. Red line, case 1; blue line, case 2; green line, case 3

During three-dimensional clock motion, the normal and reconstructed ATFLs exhibited the maximal strain value (100) during supination (Fig. 4). Although the normal ATFL was not strained when in pronation, the reconstructed ATFL exhibited strain (relative strain values of 16–36) during pronation.

**Fig. 5:**
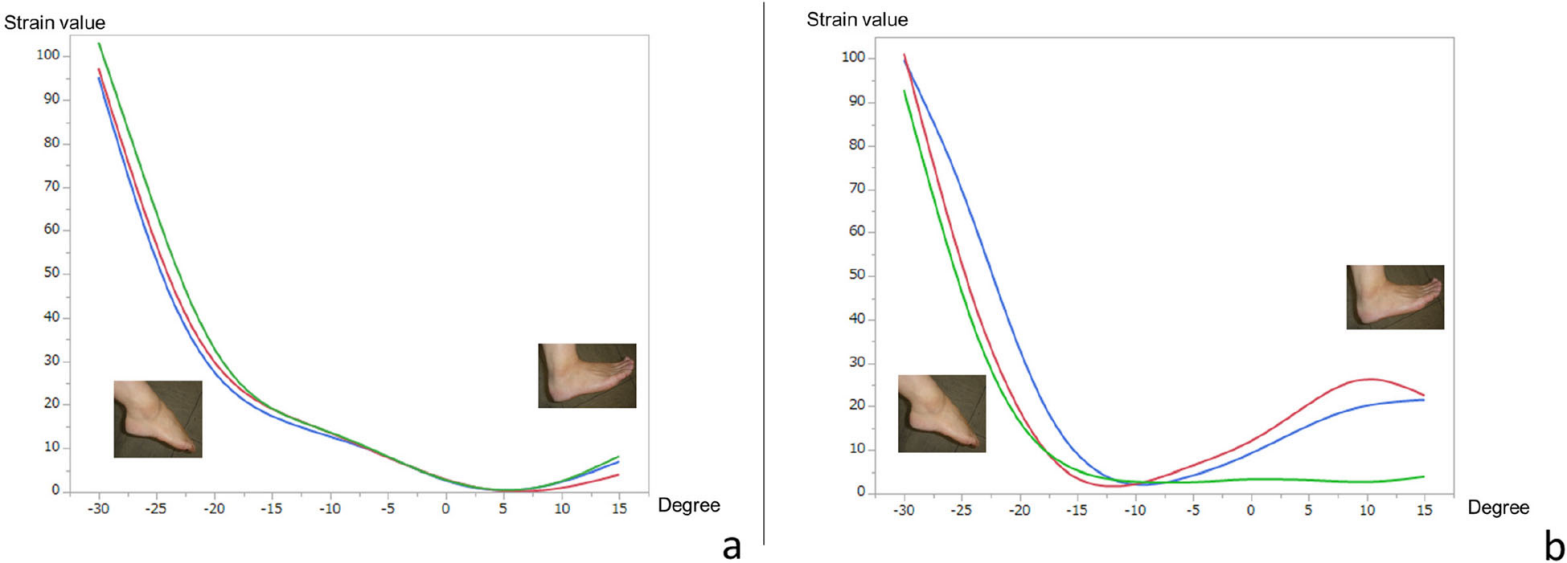
Strain patterns of the normal and the reconstructed anterior talofibular ligaments (ATFLs) during the axial motion. **a** The normal ATFL starts to gradually tense at 0° plantar flexion, with the strain increasing as the plantar flexion angle increases to the maximum strain value (100) at 30° plantar flexion. **b** The reconstructed ATFL starts to gradually tense at 15° plantar flexion, with strain increasing as the plantar flexion angle increases to the maximum strain value (100) at 30° plantar flexion. Red line, case 1; blue line, case 2; green line, case 3

During the axial motion of the ankle from 15° dorsiflexion to 30° plantar flexion, the normal ATFL started to gradually exhibit strain at 0° plantar flexion, with the strain increasing as the plantar flexion angle increased to the maximum value (strain value of 100) at 30° plantar flexion (Fig. 5a). The reconstructed ATFL started to tense gradually at 15° plantar flexion, and the strain increased as the plantar flexion angle increased to the maximum strain value (100) at 30° plantar flexion. Two specimens showed strain values of 20 and 30 at 15° of dorsiflexion (Fig. 5b).

### Strain patterns in normal and reconstructed CFLs

**Fig. 6:**
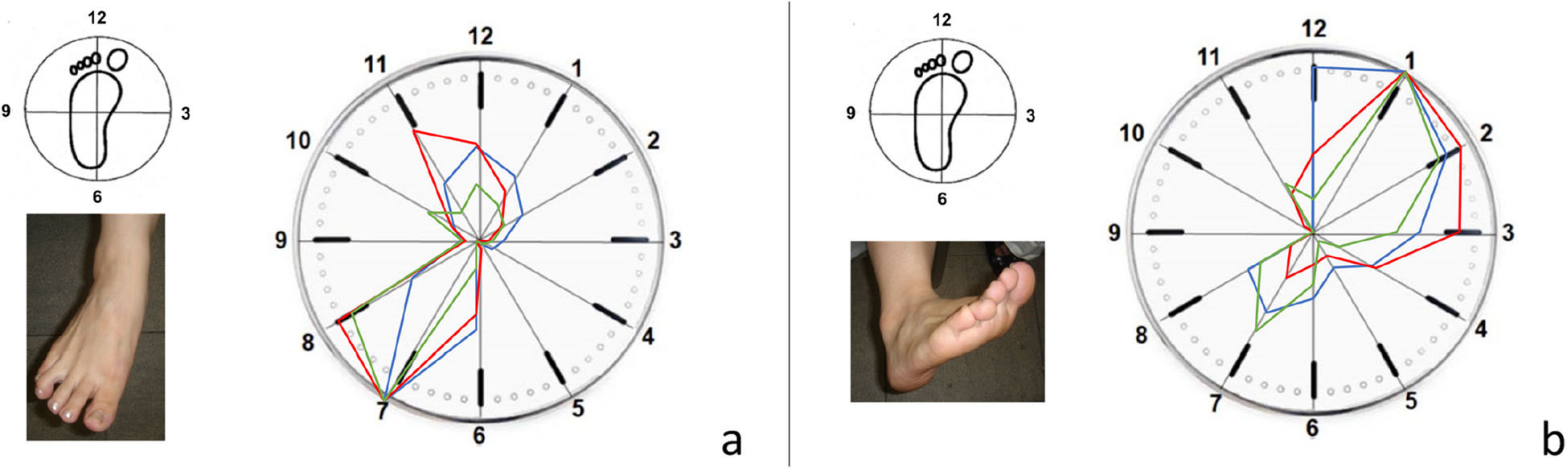
Strain patterns of normal and reconstructed calcaneofibular ligaments (CFLs) during three-dimensional motion. **a** The normal CFL exhibits the most strain during plantar flexion-abduction, with strain values of 30–52 during dorsiflexion. **b** The reconstructed CFL exhibits the most strain in dorsiflexion-adduction, with strain values of 29–62 during plantar flexion-abduction. Red line, case 1; blue line, case 2; green line, case 3

During three-dimensional motion, the normal CFL was under the most strain during plantar flexion-abduction and exhibited less strain (30–52) during dorsiflexion (Fig. 6a). The reconstructed CFL was under the most strain during dorsiflexion-adduction and exhibited less strain (29–62) during plantar flexion-abduction, which was opposite of the normal strain pattern (Fig. 6b).

**Fig. 7:**
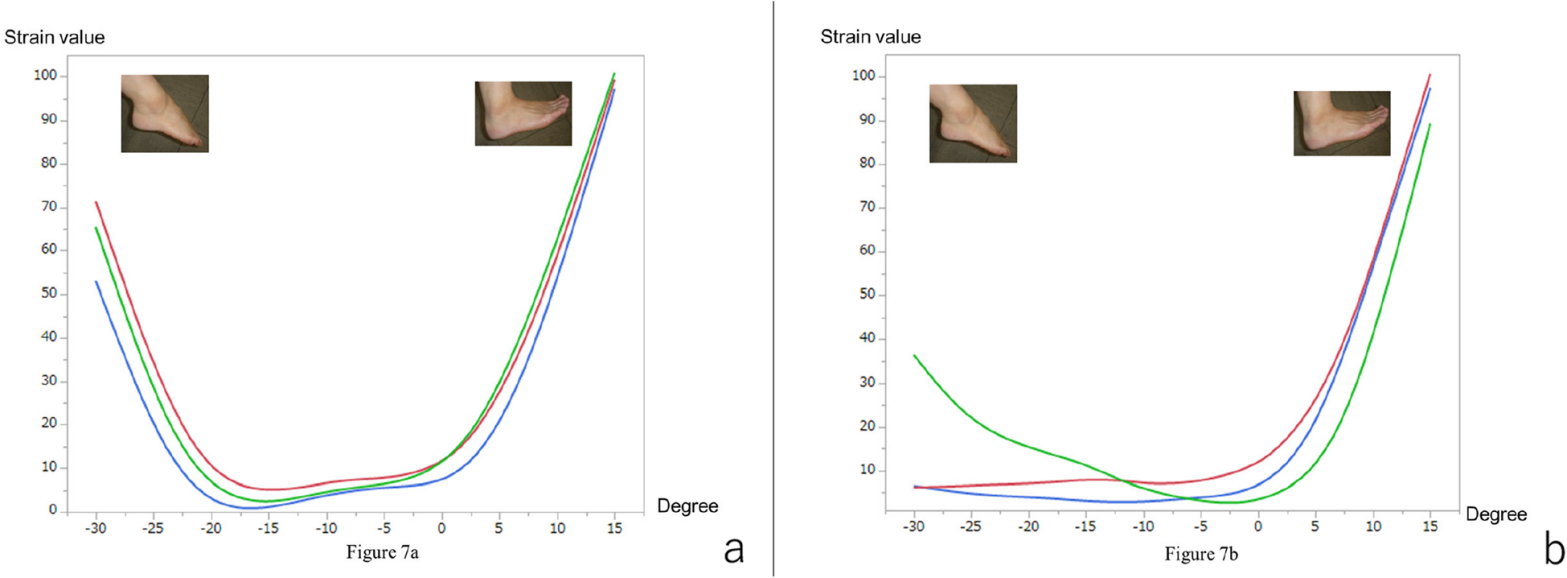
Strain patterns of normal and reconstructed calcaneofibular ligaments (CFLs) during the axial motion. **a** The normal CFL begins to gradually tense at 20° plantar flexion and 5° dorsiflexion. The strain increases as the plantar flexion and dorsiflexion angles increase. The maximum strain value (100) is observed at 15° dorsiflexion, with a relative strain value of 70 at 30° plantar flexion. **b** The reconstructed CFL begins to gradually tense at 0° dorsiflexion, with the strain increasing as the dorsiflexion angle increases; the maximum strain value (100) is observed at 15° dorsiflexion. Two specimens did not show any strain and one exhibited a strain value of 150 during plantar flexion. Red line, case 1; blue line, case 2; green line, case 3

During the axial motion, the normal CFL started to gradually tense at 20° plantar flexion and 0° dorsiflexion. The strain increased as the plantar flexion or dorsiflexion angle increased to the maximum strain value (100) at 15° dorsiflexion or strain values of 55–70 at 30° during plantar flexion (Fig. 7a). The reconstructed CFL started to gradually tense at 0° dorsiflexion, increasing as the dorsiflexion angle increased to the maximum strain value (100) at 15° dorsiflexion (Fig. 7a). Unlike the normal CFL, the two reconstructed specimens showed either no strain or a strain value of 35 during plantar flexion (Fig. 7b).

## Discussion

This is the first study to comprehensively describe the contributions of reconstructed ATFLs and CFLs to overall ankle stability in various ankle positions and to compare their tensile patterns with those of normal ATFLs and CFLs.

A previous study evaluated the tensile patterns of normal ATFLs and CFLs during three-dimensional motion and reported that the maximal tensile force in the ATFL was observed during supination with plantar flexion and that in the CFL occurred during pronation with plantar flexion [22]. These findings are similar to the present findings considering that the terms “supination with plantar flexion” and “pronation with plantar flexion” used in the previous study are analogous to our “supination” and “plantar flexion-abduction” terminologies, according to proposed terminologies [21].

During anatomical reconstruction of the lateral ankle ligament, bone tunnels are created at the anatomic origin and insertion footprints of the ligament to fix the tendon graft [12–18], thus recreating the normal anatomy. In this study, the strain patterns of the reconstructed ligaments were not always the same as those of the native, non-reconstructed ligaments. For example, the normal ATFL was not strained during pronation, whereas the reconstructed ATFL demonstrated slight strain, and the normal CFL experienced the most strain during plantar flexion-abduction, but the reconstructed ATFL exhibited the most strain during dorsiflexion-adduction. These differences in strain patterns may be attributed, in part, to slight anatomical variations that are introduced when a common origin site is used (as in the present study) as the origin of the fibular stem of the anatomic Y-graft and as the origin of both the ATFL and CFL, as previously described [18] (Fig. 3a).

If the analogous surgical procedure involving the knee is considered, anatomic anterior cruciate ligament (ACL) reconstruction is defined as the functional reconstruction of the ACL to its native dimensions, collagen orientation, and insertion site [23]. This is similar to the anatomical reconstruction of the lateral ankle ligaments, wherein a common origin site is used as the fibular stem of the anatomic Y-graft bone tunnel that is at a position midway between the original ATFL and CFL footprints.

In a prior study, the landmarks used to define the centers of origin and insertion points for the ATFL and CFL demonstrated variability [24]. Further, each footprint has an elliptical shape that varies in area and location during each surgery.

Another explanation for the variability in strain patterns between the normal and reconstructed lateral ankle ligaments may be related to the natural variations in the lengths and bundle patterns of the normal ligaments. For example, cadaveric studies have revealed that the ATFL may exist as a single bundle (31–38% of specimens), two bundles (50–60%), or three bundles (9–12%) [25, 26]. A single-bundle reconstruction technique was adopted in this study and is used in most anatomical reconstruction techniques; multiple band anatomical reconstruction techniques are no longer used. Another concern is that the normal ATFL and CFL work co-operatively with the lateral talocalcaneal ligament, and the three ligaments have a single-unit shape [27, 28]; however, the ATFL and CFL are reconstructed as solitary ligaments. In addition, the gracilis tendon used for reconstruction is different in size and shape from the actual ligament; therefore, it is difficult to completely normalize tension patterns and functions.

According to a study conducted by Brockett and Chapman [29], the range of motion of the ankle in the sagittal plane is between 65° and 75°, moving from 10°–20° of dorsiflexion to 40°–55° of plantar flexion. During daily activities, the range of motion required in the sagittal plane is much less, with a maximum of 30° for walking and 37° and 56° for ascending and descending stairs, respectively. The ATFL and CFL reconstruction in this study was performed by a technique that is similar to that used *in vivo* to fix the graft with an interference screw applying 30-N tension force [18]. Accordingly, the time zero range of motion of the ankle should be restored to dorsiflexion 15° and plantar flexion 30° similar to that *in vivo*. Therefore, this study only reflects a part of the daily routine activities.

The ultimate goals of reconstructive surgeries are to diminish pain and improve function, and previous reports have shown good clinical results after conventional anatomical reconstruction of the ATFL and CFL [12, 13]. Therefore, the goal of restored biomechanics may not be essential, but the development of a reconstructive surgery technique that aims to restore more normal biomechanics should be considered.

### Limitations

The small sample size of this study is a potential source of error. The methods described for this study indicated that the measurements for one limb required one full day to complete. Regardless, an increased sample size would reduce the associated error.

## Conclusions

This study demonstrated the biomechanical properties of each ligamentous band of normal and anatomically reconstructed ATFLs and CFLs. The results showed that the strain patterns of reconstructed ATFLs and CFLs were not exactly the same as for the normal ligaments. These findings provide a biomechanical framework for future studies aimed at improving ankle biomechanics following stabilization surgeries that seek to diminish pain and improve function in the ankles of patients with chronic ankle instability.

## Data Availability

The datasets generated during and analyzed during the current study are not publicly available due to privacy and ethical concerns but are available from the corresponding author on reasonable request.

## Abbreviations

ACL: Anterior cruciate ligament
ATFL: Anterior talofibular ligament
CFL: Calcaneofibular ligament
MLPP: Miniaturization Ligament Performance Probe
